# Chylomicron Retention Disease in A Male Infant: A Rare Case from Pakistan

**DOI:** 10.7759/cureus.7184

**Published:** 2020-03-05

**Authors:** Sohail Kumar, Deedar Nanjiani, Faryal Tahir, Dua Azim, Oam Parkash

**Affiliations:** 1 Internal Medicine, Dow Medical College and Dr. Ruth K. M. Pfau Civil Hospital Karachi, Karachi, PAK; 2 Internal Medicine, Dow University of Health Sciences, Karachi, PAK; 3 Pediatrics, Dow University of Health Sciences, Karachi, PAK

**Keywords:** chylomicron retention disease, anderson disease, hypocholesterolemia, sar1b gene, autosomal recessive, fat malabsorption, chylomicrons, steatorrhea, snowflake appearance, fat-soluble vitamin deficiency

## Abstract

Chylomicron retention disease (CMRD), also known as Anderson’s disease, is an autosomal recessive condition with a genetic mutation in the secretion associated Ras related GTPase 1B (SAR1B) gene, a protein coding gene. CMRD classically manifests as steatorrhea, vomiting, failure to thrive or abdominal bloating shortly after birth or in childhood. Here, we report a rare case of a 50-day-old male infant who was, at first, overseen as a case of acute gastroenteritis with sepsis owing to the non-specific symptoms i.e. multiple episodes of loose stools with a low-grade fever and failure to thrive, and was managed accordingly. However, the symptoms did not resolve; moreover, the clinical condition deteriorated. Later, lipid profile, clinical presentation and pathological features led to a presumptive diagnosis of CMRD. Our patient showed significant improvement when treated with a trial of medium- and short-chain fatty acids. We conclude that, in resource-restricted countries, a therapeutic trial with the dietary changes is essential to not only prevent the devastating complication but also support the diagnosis.

## Introduction

Chylomicron retention disease (CMRD), also called Anderson's disease (AD), is a rare hereditary disorder of familial hypocholesterolemia associated with the deficiency of apolipoprotein B-48 (Apo B-48). It is an autosomal recessive disorder with a prevalence of less than one per million individuals. The disease was first described by Anderson in 1961 [1]. In 2003, mutation in the secretion associated Ras related GTPase 1B (SAR1B) gene on chromosome 5 was identified in the pathogenesis of CMRD [2]. These mutations disable the formation of coat protein complex II (COPII) and, thus, block the transport of chylomicrons from the endoplasmic reticulum (ER) to the Golgi complex resulting in an increased deposition of lipids in the mucosal cells of the duodenum and, hence, decreased concentrations of cholesterol in the blood [3].

Patients usually present in infancy with non-specific complaints of steatorrhea, chronic diarrhea, vomiting, abdominal distension and failure to thrive. Consanguinity is frequent in CMRD. Patients may also show signs of fat-soluble vitamin deficiency such as ocular defects, neurological impairment and coagulation problems. Due to such non-specific symptoms, diagnosis is often delayed accompanied with a non-compliance to follow up especially in the developing or underdeveloped countries including Pakistan. Therefore, the clinical condition worsens.

We report a rare case of a 50-day-old male infant who presented in the emergency department (ED) with primary complaints of loose stools and failure to thrive. Laboratory investigations showed decreased levels of fat-soluble vitamins, low serum concentrations of low-density lipoprotein (LDL), high-density lipoprotein (HDL) and total cholesterol (TC) but normal triglycerides (TG). He was presumably diagnosed as a case of CMRD on the basis of guidelines made by Peretti et al. [4]. Treatment with low-fat diet (LFD) and fat-soluble vitamin supplementation was started, and the patient showed marked improvement in his symptoms.

## Case presentation

A 50-day-old male infant presented in the ED with complaints of loose stools since birth, fever for the last four days and failure to thrive. The frequency of stools was reported to be approximately 10-12 episodes per day. Oily and foul smelling diarrhea was yellow to green in color and did not contain blood. It was not associated with vomiting but the frequency increased with time. The fever was low grade, intermittent and temporarily relieved by antipyretics. The patient was the twelfth product of a consanguineous marriage and was a full-term pregnancy delivered by caesarean section (C-section) due to previous C-section scars. Birth weight of our patient was 2 kg. There were no antenatal or postnatal complications except that he was admitted for three days on 35th day of life for the same complaints, and was managed accordingly. He had exclusively been on breastfeed. He achieved social smile at six weeks. There was a history of death of his sibling at the age of one year secondary to loose stools.

Upon examination, patient's body weight was found to be 2.5 kg (<2nd percentile), body length was 49.5 cm (<1st percentile) and the occipital-frontal head circumference was 35 cm (<1st percentile). He was found lethargic, dehydrated, anemic and febrile with a distended abdomen and signs of failure to thrive. Further examination revealed extensive perianal rash, oral ulcers and overriding sutures. All other systemic examinations were unremarkable.

Laboratory investigations revealed low levels of hemoglobin, normal mean corpuscular volume and mean corpuscular hemoglobin but increased total leukocyte count (Table 1). The patient was initially managed as a case of acute gastroenteritis with sepsis owing to leukocytosis and pus cells with fat globules in the stool detailed report. Therefore, intravenous fluids and antibiotics were administered. This showed an improvement of the hydration status and the fever was subsided, but loose stools remained the same. Furthermore, the clinical condition worsened on feeding. The patient was then investigated for other possible causes of infantile diarrhea. The probable differential diagnosis for malabsorption are lactose intolerance (LI), congenital chloride losing diarrhea (CCLD), abetalipoproteinemia (ABL) and CMRD. In order to exclude infectious causes of chronic diarrhea, stool and blood cultures were ordered which showed no growth. Absence of reducing substance in stool test and normal levels of serum electrolytes excluded LI and CCLD, respectively. Furthermore, lipid profile was ordered which showed low levels of LDL, HDL and TC, whereas TG levels were within normal range. These findings excluded ABL as well. The plasma LDL and TC levels of his parents were within normal range, and they did not change significantly after a fat challenge. Low serum concentrations of fat soluble vitamins i.e. vitamin A, D and E were found. Prothrombin time and international normalized ratio were increased which reflected deficiency of vitamin K. Liver function test revealed raised alkaline phosphatase levels with normal aspartate aminotransferase, alanine aminotransferase, total bilirubin and gamma-glutamyl transpeptidase values. Serum albumin concentration was found to be decreased and a subsequent decrease in the albumin-globulin ratio was observed (Table 1).

**Table 1:** Laboratory Investigations of the Patient Hb, hemoglobin; Hct, hematocrit; MCV, mean corpuscular volume; MCH, mean corpuscular hemoglobin; MCHC, mean corpuscular hemoglobin concentration; TLC, total leukocyte count; PLT, platelet; CRP, C reactive protein; LDL, low-density lipoprotein; HDL, high-density lipoprotein; TC, total cholesterol; TG, triglycerides; PT, prothrombin time; aPTT, activated partial thromboplastin time; INR, international normalized ratio; ALP, alkaline phosphatase; AST, aspartate aminotransferase; ALT, alanine aminotransferase; T-Bil, total bilirubin; GGT, gamma-glutamyl transpeptidase; A/G ratio, albumin to globulin ratio.

Laboratory Investigation	Patient’s Value	Reference Range
Hb	9.5	13-17 g/dL
Hct	27	40%-50%
MCV	84	80-100 fL
MCH	24	27-32 pg
MCHC	34	31.5-34.5 g/dL
TLC	20000	4000-10000/μL
Neutrophils	43	40%-75%
Lymphocytes	41	20%-45%
PLT	219000	150000-450000/μL
CRP	42	<3 g/dL
LDL	16	34-131 mg/dL
HDL	16	23-60 mg/dL
TC	60	94-190 mg/dL
TG	137	38-198 mg/dL
Vitamin A	155	194-421 µg/L
Vitamin D	43.7	52-250 nmol/L
Vitamin E	1.98	3.02-9.05 mg/L
PT	25.9	9-12 s
aPTT	31.5	30-45 s
INR	2.4	0.7-1.2
ALP	142	40-112 U/L
AST	45	9-80 U/L
ALT	19	13-45 U/L
T-Bil	0.46	0-1 mg/dL
GGT	58	13-147 U/L
Albumin	2.6	3.5-5 g/dL
Globulin	3.4	2-3.5 g/dL
A/G ratio	0.76	1.2-1.5

The findings were suggestive of CMRD. The facility of colonoscopy and biopsy was not available at our setup. A presumptive diagnosis of CMRD was made on the basis of guidelines made by Peretti et al. [4]. In order to confirm the diagnosis and, thereby, treat our patient, we put him on a trial of medium-chain (MCFA) and short-chain fatty acids (SCFA). As expected, our patient’s clinical condition improved markedly which was reflected by a noticeable fall in the frequency of diarrhea i.e. from 10 episodes per day to two episodes daily, within a week. After being clinically stable, our patient was discharged with this LFD to which he showed gradual improvement over the next year. His body weight increased up to 45th centile as well as the diarrhea subsided. He successfully achieved his developmental milestones and was also kept on fat-soluble vitamin supplementation (hydrosoluble vitamin E: 50 UI/kg/d, vitamin A: 15,000 IU/d, vitamin K: 15 mg/wk, vitamin D: 800-1,200 UI/kg/d) under close monitoring to ensure normal levels and prevent toxicities.

## Discussion

Previously known as AD, CMRD was first described by Anderson, in 1961, in a seven-month-old child with persistent neonatal diarrhea [1]. However, the term "CMRD" is usually preferred because the name indicates an underlying defect. Chylomicrons are lipoproteins that are composed of a lipid core surrounded by a coat of phospholipids and proteins, secreted exclusively from the intestine as the nascent chylomicrons. They transport lipids to all the tissues in the body and are then taken up by the liver as chylomicron remnants, where they are endocytosed and degraded. Apo B-48 is a protein unique to chylomicrons. There is a specific decrease in the levels of Apo B-48 in the postprandial state.

Many collaborative studies and genetic screening have identified the culprit gene, SAR1B, responsible for CMRD [2]. SAR1B gene, located on chromosome 5q31.1, is affected by different types of mutations such as frameshift, splice site and missense mutation. As a result, it disorganizes the protein product which later forms part of the vesicular COPII [5]. These defective SAR1B proteins result in a failure to transport chylomicrons from ER to Golgi complex [6]. To date, about 20 mutations have been reported in roughly 50 patients [7-10]. SAR1B gene is also responsible for the secretion of very low density lipoproteins from hepatocytes.

CMRD classically manifests as steatorrhea, vomiting, failure to thrive or abdominal bloating shortly after birth or in childhood which is similar to the clinical picture of our patient. Although diarrhea is universally present in all CMRD patients, only one case has been reported of CMRD without diarrhea [11]. Other manifestations of CMRD due to fat malabsorption include fat-soluble vitamin deficiency that could lead to serious complications such as ophthalmopathy owing to vitamin A deficiency, osteopenia and rickets due to vitamin D deficiency, and neurological complaints secondary to the deficiency of vitamin E. Therefore, vitamin E deficiency is the most important and troublesome among all, because its transport is highly based on Apo-B containing lipoproteins and its deficiency causes degenerative nerve disorder such as proprioceptive abnormalities, polyneuropathy and ataxia [4]. However, these complications appear more often in long-standing undiagnosed cases. Myopathy, hepatomegaly and hepatic steatosis have been reported in some cases of CMRD [12].

As seen in our case, diagnosis of CMRD is very challenging and often delayed owing to its non-specific symptoms that may resemble relatively common conditions, such as food allergies. It is often confused with other genetic disorders, such as ABL, and those with decreased LDL levels, such as homozygous hypobetalipoproteinemia. Thus, identification of the SAR1B gene mutation through genetic testing is necessary for a confirmatory diagnosis [2]. Clinical symptoms of malabsorptive chronic diarrhea, failure to thrive and an abnormal lipid profile with more than 50% decrease in TC, LDL and HDL cholesterol with normal levels of TG are highly suspicious and pathognomic features of CMRD. In our case and in other resource-restricted nations, where genetic testing is not readily available, a presumptive diagnosis could be made on the basis of deranged lipid profile, clinical presentation and pathological features as described in the guidelines proposed by Peretti et al. [4]. Endoscopy supports the diagnosis by showing a normal esophageal and gastric mucosa with marked whitening of duodenal mucosal layer, a pattern often described as "snowflake appearance." This pattern is attributed to the accumulation of fat within the cells of gastric mucosa and, hence, such cells are called "fat-filled enterocytes.'' Biopsy, generally, shows normal villi, but sometimes mild atrophy can be found [13]. Unfortunately, due to limited resources and unavailability of these investigations at our clinical setup, we could not perform colonoscopy and biopsy.

Management of CMRD is focused on the early detection and prevention of long-term devastating complications. Thus, treatment aims at optimizing absorption and growth by removing long-chain fatty acids from the diet as well as preventing nutritional deficiencies via administering vitamin supplements, most importantly vitamin E supplements as we did in our case [4]. Early treatment with SCFA and MCFA containing diet and fat-soluble vitamins in patients with suspected CMRD, following exclusion of other probable causes, is not only essential to prevent and minimize devastating complications but also to support the diagnosis. A trial of MCFA diet showed a spectacular improvement in our patient’s outcome as evident by a significant decrement in the episodes of diarrhea within a week. Our patient was also put on fat-soluble vitamin supplements as they are an essential part of management to avoid complications [4].

Despite the fact that the patient's gastrointestinal (GI) symptoms show a significant improvement following an LFD, diarrhea begins whenever fat is reintroduced in the diet, even in the adult population. Furthermore, there is no improvement in steatorrhea after an average of five years of follow-up [14]. This suggests that if low-fat regime is not followed, malabsorption can impede growth in infancy. Hence, it is necessary that, during childhood, annual follow-ups should be performed with clinical examinations and biological evaluations focusing on nutritional growth and GI, hepatic and neurological manifestations and complications to encourage better prognosis.

Our purpose is to emphasize on the approach that early treatment with medium-low chain fat diet and fat-soluble vitamin supplementation should be considered in patients suspected of CMRD, when genetic testing is not feasible for corroborative diagnosis, especially in countries with meager resources. This prevents further damage to the patients and also supports the diagnosis. Thus, management ought to be coordinated towards prevention and early recognition of complications. Focus on dietary counseling to maintain adequate caloric and essential fatty acid intake and regular follow-ups to closely observe treatment compliance together with monitoring nutrition and development should be encouraged for better prognosis.

## Conclusions

CMRD is a rare familial hypocholesterolemic syndrome characterized by lipid malabsorption. Due to its rarity, more research is required to better understand its pathophysiology and contributing factors. Owing to its non-specific symptoms, the diagnosis can easily be overseen. Although rare, hypocholesterolemic disorders should be suspected in infants who present with vomiting, diarrhea and failure to thrive. Moreover, a timely diagnosis along with therapeutic trial with dietary changes is essential for a better outcome and helps prevent serious clinical complications. Early supplementation with fat-soluble vitamins should also be considered in these patients, as it can further prevent risk of clinical deterioration.
